# Plasmacytoid Urothelial Carcinoma of the Bladder That Manifests Disseminated Carcinomatosis of the Bone Marrow: A Case Report of Extremely Rapid Progression

**DOI:** 10.1155/2022/6082700

**Published:** 2022-09-15

**Authors:** Nobuhiko Shimizu, Yoshinobu Moritoki, Nao Katsumi, Takahiro Yanase, Teruaki Sugino, Kazuhiro Kanemoto, Hidetoshi Akita, Takahiro Yasui

**Affiliations:** ^1^Department of Urology, Anjo Kosei Hospital, Anjo, Japan; ^2^Department of Urology, Toyokawa City Hospital, Toyokawa, Japan; ^3^Department of Nephro-Urology, Nagoya City University Graduate School of Medical Sciences, Nagoya, Japan

## Abstract

Plasmacytoid urothelial carcinoma (PUC) of the bladder is a rare variant of invasive urothelial carcinoma (UC) with aggressive behavior. Despite its prognosis being poorer than that of conventional UC, a median overall survival of approximately 2 years is ensured when it is treated with radical cystectomy (RC), and few patients die within a few months of RC. In this paper, we report the case of a patient with PUC who developed widespread bone metastasis only 6 weeks after RC, which resulted in death within 2 months postoperatively.

## 1. Introduction

Plasmacytoid urothelial carcinoma (PUC) is a rare variant of invasive urothelial carcinoma (UC) and accounts for 1% to 3% of all invasive UCs [1]. Compared to conventional UC, PUC has a greater chance of higher-stage disease, metastasis at presentation, and poor prognosis [2]. Although clinical data on the prognosis of patients with PUC are limited, their median overall survival when treated with radical cystectomy (RC) and adjuvant cisplatin-based chemotherapy is 27.4 months (95% CI: 16.8-37.9 months) [3], and few patients die within a few months of RC. In this paper, we report a case of PUC that developed widespread bone metastasis 6 weeks after RC, which resulted in death within 2 months postoperatively.

## 2. Case Presentation

**Figure 1:**
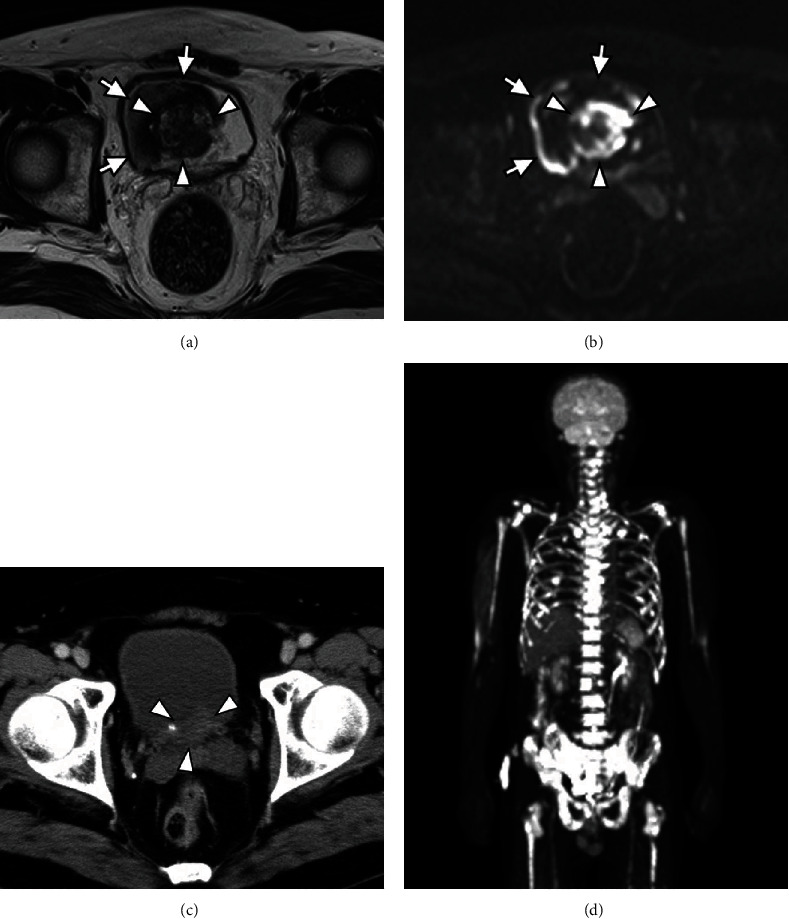
(a) MRI T2-weighted image and (b) diffusion-weighted image showed a bladder tumor (white arrowheads) with hematoma (white arrows). (c) The CT image after NAC did not show rapid progression of the bladder tumor (white arrowheads). (d) The FDG-PET findings when the patient presented with low back pain after the RC.

**Figure 2:**
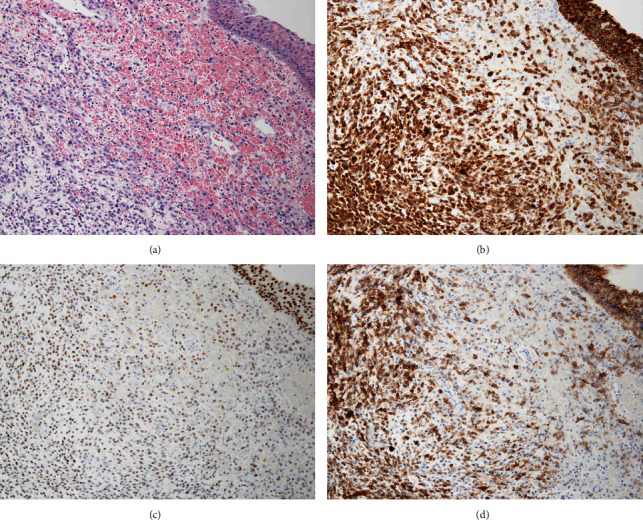
(a) HE staining of the tumor when transurethral electrocoagulation was performed. (b–d) Immunohistochemical staining findings of the tumor for pancytokeratin (b), GATA3 (c), and CD138 (d) when transurethral electrocoagulation was performed.

**Figure 3:**
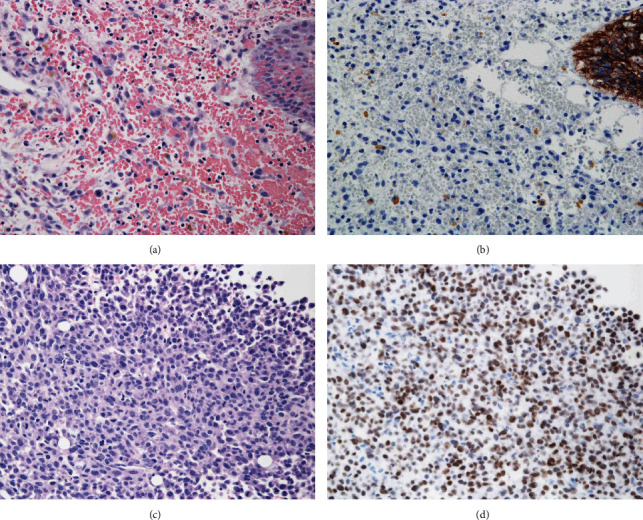
(a) HE staining and (b) immunohistochemical staining for E-cadherin expression of the tumor when transurethral electrocoagulation was performed. (c) HE staining and (d) immunohistochemical staining for GATA3 from the second bone marrow biopsy.

A 56-year-old man with hypertension and dyslipidemia was admitted to the hospital because of urinary bladder clot retention. The patient was asymptomatic until admission. He had smoked 54 pack-years. Magnetic resonance imaging (MRI) revealed a tumor and a hematoma in the bladder (Figures 1(a) and 1(b)). On account of continuous hemorrhage and anemia, transurethral electrocoagulation and resection of a nonpedunculated bladder tumor were performed. Hematoxylin and eosin (HE) staining of the bladder tumor showed discohesive malignant cells with eccentrically placed nuclei and abundant cytoplasm, resembling plasma cells. The resection specimen did not contain bladder muscle (Figure 2(a)). The tumor cells were immunoreactive for pancytokeratin (Figure 2(b)), GATA3 (Figure 2(c)), and CD138 (Figure 2(d)), thereby leading to the diagnosis of invasive PUC, grade 3 (G3), pT1, or higher. Immunohistochemical staining for E-cadherin expression in PUC cells was negative, which suggested an aggressive phenotype of this bladder tumor (Figures 3(a) and 3(b)). Computed tomography (CT) did not show any metastasis in the lymph nodes or other organs, and MRI did not show any muscle invasion. Since upstaging of pT1 tumors diagnosed with transurethral resections is common, RC was performed, after three cycles of neoadjuvant chemotherapy (NAC) with gemcitabine and cisplatin. CT imaging obtained after NAC did not show rapid progression of the bladder tumor (Figure 1(c)). The pathological stage was pT3aN0 and RM0 with lymphovascular invasion. The postoperative course was uneventful, although the patient presented with low back pain on the 39^th^ postoperative day. The hematologic examination on admission revealed a high white blood cell count (129,600 cells/*μ*L), high serum alkaline phosphatase (1039 U/L), hypercalcemia (17.0 mg/dL), and low serum albumin level (3.3 g/dL). These findings are indicative of bone diseases such as primary myeloproliferative disease or bone metastasis. The ^18^F-fluorodeoxyglucose (FDG) positron emission tomography (PET) findings revealed significant FDG uptake in nearly all bones (Figure 1(d)). Histological findings of the bone marrow biopsy were negative for malignancy, which seemed to be a pseudonegative finding.

On the 53^rd^ postoperative day, the patient passed away, which occurred just after a second bone marrow biopsy had been performed. Histological findings of the biopsy showed metastasis of the PUC (Figures 3(c) and 3(d)). The autopsy revealed metastasis of the bone marrow concomitant with small metastasis to the lung, chest wall, and spleen, which were not detected on the FDG-PET image.

## 3. Discussion

PUC is clinically aggressive and has a worse median overall survival (27.4 months) than does conventional UC (62.6 months; *P* = 0.13) when treated with RC and adjuvant cisplatin-based chemotherapy [3]. Its prognosis is worse, although it allows for 2 years of life expectancy after RC. However, in this case, the patient died 2 months after the surgery because of the extremely rapid progression of the disease.

A previous study reported that PUC has a poor response to NAC [4], whereas a systematic review reported that NAC was beneficial for PUC [5]. Therefore, the treatment strategies for PUC remain controversial. After we discussed whether to choose NAC or upfront RC, we decided on NAC for this patient in the hope that NAC could downstage the disease.

This patient was also diagnosed as having disseminated carcinomatosis of the bone marrow (CBM), which is characterized by widespread bone metastasis from solid tumors with coexistent hematologic disorders [6]. CBM is most frequently seen as a complication of gastric cancer, and only one case has been reported with urothelial carcinoma [7]. Despite the multidisciplinary treatment, the prognosis of CBM is quite poor (less than 1 year of life expectancy). In this patient, the prognosis was only within 2 months.

Investigators have noted that loss of E-cadherin expression may be associated with a plasmacytoid differentiation pattern in bladder UC, thereby explaining the aggressive behavior of PUC [1]. E-cadherin loss is associated with loss of cell differentiation and increased cell invasion, which leads to more aggressive pathology and poor prognosis in cases of bladder UC [8]. The effect of E-cadherin loss on the prognosis of PUC has not been established, although the complete loss of membranous E-cadherin expression was observed in as many as 76.2% of PUC cases [9]. In this patient, the tumor showed complete loss of membranous E-cadherin expression (Figure 3(b)), which may explain the poor prognosis in this patient.

According to previous studies [10, 11], an advanced pathological stage on RC after NAC and lymphovascular invasion is associated with a poor prognosis. Loss of E-cadherin, disseminated CBM, an advanced pathological stage after NAC, and lymphovascular invasion may have contributed to the extremely low life expectancy. A fact that must be noted is that some cases of PUC progress rapidly, even when the presurgical stage is non-muscle-invasive UC.

## Data Availability

The data that support the findings of this case report are available from the corresponding author, Yoshinobu Moritoki, upon reasonable request.
